# A meta-analysis: retrograde intrarenal surgery vs. percutaneous nephrolithotomy in children

**DOI:** 10.3389/fped.2023.1086345

**Published:** 2023-05-02

**Authors:** Yi Yuan, Yan-nei Liang, Kai-feng Li, Yi-ru Ho, Qian-long Wu, Zhang Zhao

**Affiliations:** ^1^ Department of Pediatric Urology Surgery, Guangzhou Women and Children's Medical Center, Guangzhou Medical University, Guangdong Provincial Clinical Research Center for Child Health, Guangzhou, China; ^2^ Department of Urology Surgery, Guangzhou Women and Children’s Medical Center of Guangzhou Medical University, Guangzhou, China

**Keywords:** nephrolithotomy, percutaneous, retrograde intrarenal surgery, child, kidney calculi, meta-analysis

## Abstract

**Backgrounds:**

The increasing prevalence of pediatric kidney stones worldwide makes minimally invasive lithotripsy like retrograde intrarenal surgery (RIRS) and percutaneous Nephrolithotomy (PCNL) more prevalent. However, their safety and efficacy are controversial. Consequently, a meta-analysis of the comparison between RIRS and PCNL is conducted.

**Methods:**

Clinical trials were selected from PubMed, EMBASE, Scopus, and Cochrane Library databases. The data extraction and study quality assessment were performed by two individuals independently. The data relating to therapeutic effects were extracted and analyzed by Review manager 5.4.

**Results:**

Thirteen studies involving 1,019 patients were included. The micro-PCNL excelled in stone-free rate (*P* = 0.003), postoperative fever rate (*P* = 0.02), and Clavien–Dindo II complications (*P* = 0.05). Notably, the mean age of the micro-PCNL group was younger than other groups (*P* = 0.0005). The operation time in mini-PCNL was longer than RIRS (*P* < 0.00001) but with high heterogeneity (*I*^2^ = 99%). There was no difference in Clavien–Dindo I, II, and III complications between the PCNL and the RIRS, but mini-PCNL showed a higher probability than RIRS in Clavien–Dindo I (*P* = 0.0008) and II complications (*P* = 0.007).

**Conclusions:**

Compared with RIRS, micro-PCNL could be a better therapeutic option for kidney stones in children. Of note, more parameters should be analyzed to illustrate the efficacy of different minimally invasive surgeries for pediatric kidney stones due to poor cases in our study.

**Systematic Review Registration:**

https://www.crd.york.ac.uk/prospero/#recordDetails, PROSPERO CRD42022323611

## 1. Introduction

Previous epidemiological studies have shown that the kidney stone incidence rate has risen. Besides, we found that pediatric patients were at high risk for kidney stone disease. Meanwhile, the risk of kidney stones in younger children was related to diabetes mellitus and hypertension. Simultaneously, the little girls likely suffered from kidney stone disease (1, 2).

RIRS (Retrograde Intrarenal Surgery, RIRS) is one of the primary measures to dispose of kidney stone disease in children. Compared with PCNL, RIRS has the advantage that it uses a natural orifice, which t requires no additional pathway for lithotripsies. For a child who suffers from kidney stones, RIRS can be a good option as the entrance of RIRS surgery is a natural pipeline of the human body which is smaller, the treatment is safer, and RIRS is more conducive to postoperative recovery (3). Nevertheless, during the operation of the RIRS for kids, the usage of sizeable medical instruments and the low-quality optics technology raise the possibility of the development of ureter ischemia, ureter injury, ureter stenosis, and reflux from the bladder and ureter (3).

PCNL is a frequently-used therapeutic option for pediatric kidney calculi. There are various types of PCNL, including standard PCNL (24–30 Fr), mini-PCNL (16–18/20 Fr), ultra-mini PCNL (11–14 Fr), micro-PCNL (<10 Fr), etc. PCNL is suitable for more giant stones. If we select using the Mini-Micro PCNL, the instrument can enter the renal cortex under the condition of direct vision. Besides, the expansion can be avoided after entering the renal cortex. Consequently, the operative time can be shortened, the radiation exposure can be decreased, and the complications, including hemorrhage and perforation, which were connected with expanding of the urinary tract, can be averted (4, 5).

Recently, many meta-analyses have compared the RIRS and PCNL's advantages and drawbacks (40, 41). However, we find that most of these articles are not convincing enough because the clinical trials included do not enough. Therefore, we aim to conduct a meta-analysis and systematic review of published articles to illustrate the superiority of RIRS and PCNL in treating children with upper urinary stones disease. We hope to include a more significant number of relevant articles and eliminate heterogeneity. We hope our meta-analysis could provide preferable guidance for surgeons treating children kidney stones.

## 2. Materials and methods

### 2.1. Information sources

Three investigators searched the databases independently, including PubMed, Scopus, Cochrane library, and Embase, with MeSH terms and Entry terms, to single out the eligible articles based on the inclusion and exclusion standards by May 30, 2022. The MeSH words include “child,” “Nephrolithotomy”, and “Percutaneous.” Nevertheless, a part of the free term which had not to correspond with Mesh terms, including “Nephrolithotomy, Percutaneous,” “F-URS,” “URS,” “FURS,” “flexible URS,” “Retrograde intrarenal surgery,” “RIRS. “The comprehensive searching formula which we used in the PUBMED was the following: [retrograde flexible ureterorenoscopy(Title/Abstract)] OR [flexible ureterorenoscopy(Title/Abstract)] OR [F-URS(Title/Abstract)] OR [URS(Title/Abstract)] OR [FURS(Title/Abstract)] OR [Retrograde intrarenal surgery(Title/Abstract)] OR [RIRS(Title/Abstract)] OR [flexible URS(Title/Abstract)] OR [URS(Title/Abstract)] AND [Nephrolithotomy, Percutaneous(MeSH Terms)] OR [Nephrolithotomies, Percutaneous(Title/Abstract)] OR [Percutaneous Nephrolithotomies(Title/Abstract)] OR [Percutaneous Nephrolithotomy(Title/Abstract)] OR [PCNL(Title/Abstract] AND [Child(MeSH Terms)] OR [Children(Title/Abstract)] OR [Paediatric(Title/Abstract)] OR [Pediatrics(Title/Abstract)].

### 2.2. Inclusion standard and exclusion standard

Inclusion standards:

(1) The research included randomized controlled trials (RCTs) and retrospective clinical trials conducted in any country;
(2) Patients: The kids (mean age below 12 year) who suffered from kidney stone diseases;
(3) Intervening measure and comparing measure: There were at least two groups in the randomized controlled trials. The PCNL had been used in one, and RIRS had been used in another;
(4) Outcome measurement: Only if the study included at least one of the following, including stone-free rate, operative time, BMI, blood transfusion, and overall complications, we would incorporate it into the meta-analysis;
(5) The written language in English only;

Exclusion standards:

(1) Children with chronic renal failure and a history of renal calculi surgery.
(2) We will eliminate the non-controlled trials;
(3) The conference abstract, guidelines, case reports, not comparisons, review articles, and irrelevant interventions will be eliminated;

### 2.3. Data extraction

Two investigators independently conducted the data extraction procedure. The relevant information, characteristics, and results were retrieved and arranged into tables in Microsoft Excel 2019. The following data have been taken out:

(1) Stones' situation: stone size, stone location, stone density, stone masses, stone side, stone burden, stone site, stone composition, the opacity of the stones,
(2) Operation condition: operation time, lithotripsy time, fluoroscopy time, tract dilation, Double-J stent, balloon dilation of the ureteral orifice, ureteral access sheaths, imaging, irrigation fluid volume, the number of anesthesia sessions, frequency of fragmentation, laser, power, preoperative positive urine culture.
(3) Postoperative outcome: stone-free rates, complication rates, the number of complications, and their Clavien grade (postoperative fever, sepsis, postoperative hematuria, renal colic, intraoperative bleeding, hydronephrosis, urinary tract infection, urosepsis.
(4) Other variables: country, age, gender, BMI, SWL history, preoperative fever.

A third-party investigator will assess the study findings and, following sufficient deliberation, decide on an overall policy if there are differences in the data or between the findings of the two investigators.

### 2.4. Missing data

If the data recorded in the clinical trials were incomplete or led to high heterogeneity, we would conduct a sensitivity analysis, and the data were eliminated or not depending on the sensitivity analysis's result. When there were no precise sample sizes, we tried our best to estimate the sample sizes accurately. When dispersion degree in the continuous data was not reported with standard deviation (SD), but the *P*-values between two groups were presented, then these continuous data were included in our meta-analysis after the mathematical transition (6).

### 2.5. Quality assessment

Two researchers used the Cochrane handbook and the Jadad scale to evaluate the articles' quality. Furthermore, the Cochrane risk tool will assess all aspects of the risk, including selection bias, reporting bias, attrition bias, and performance bias. Besides, the assessment of the risk of bias was utilized by Review Manager V.5.4. Meanwhile, we will appraise the methodological quality of each trial and grade by using the Jadad scale. Finally, under the evaluation, if the studies can be obtained, 4 points or more will be identified as high-quality trials.

### 2.6. Data synthesis

After utilizing the Review Manager V.5.4 to analyze the inclusion trial's data, we got the mean difference (MD) for 95% CI and obtained the mean and SD from the continuous results. Also, the odds ratio (OR), which was obtained from the dichotomous data, is calculated for 95% CI. All of the data have been inspected by the random effects model. The forest plots revealed the heterogeneity of all statistical tests through the *I*^2^ value. Furthermore, it is generally considered that 1%–25%: the heterogeneity is low; 25%–50%: moderate heterogeneity; >75%: significant heterogeneity (6). To specify the efficacy of the results, sensitivity analysis, and subgroup analysis were utilized. To evaluate the risk of publication bias, we used Egger's test. Meanwhile, the *P*-value <0.05 demonstrates a statistically significant distinction between the RIRS group and the PCNL group. Ultimately, this meta-analysis was conducted under the Preferred Reporting Items for Systematic Reviews, and our study was evaluated with Meta-Analyses AMSTAR guidelines.

## 3. Results

### 3.1. Study selection

**Figure 1:**
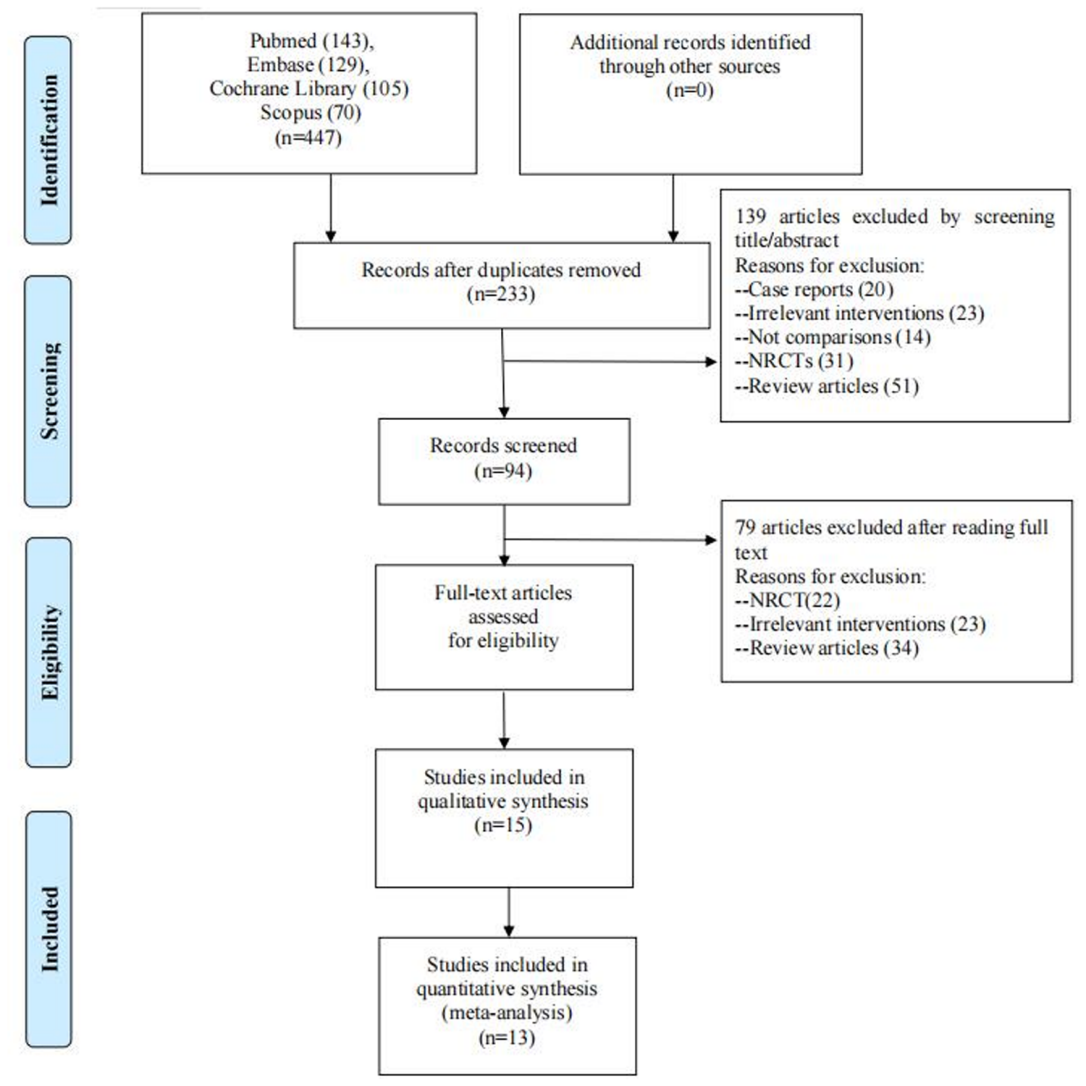
Selection flowchart of included studies.

Four hundred and forty-seven studies were collected in total, which came from PubMed (*n* = 143), Embase (*n* = 129), Cochrane Library (*n* = 105), Scopus (*n* = 70), and additional records identified through other sources (*n* = 0). After removing duplicate studies, two hundred and thirty-three researches were retained. One hundred and thirty-nine articles were excluded based on their title/abstract, including twenty case reports, twenty-three irrelevant interventions, fourteen studies without comparisons, thirty-one NRCTs, and fifty-one review articles. Additionally, seventy-nine studies were rejected because twenty-three of them had irrelevant interventions, twenty-two of them were not randomized controlled trials, and thirty-four of them were review articles. Ultimately, 13 studies were selected in our final analysis by conducting qualitative synthesis on retained articles (Figure 1) (7–19).

### 3.2. Study characteristics

Thirteen studies were selected with 1,019 patients in total. Five hundred thirty children were treated with retrograde intrarenal surgery (RIRS group), while 489 children were conducted with percutaneous Nephrolithotomy (PCNL group). The trial data were from 2008 to 2021 in Asia, Europe, and Africa. The mean age of the trial population ranged from 1.6 to 10.9 years, and the sex ratio (male/female) ranged from 19/26 to 57/16. Meanwhile, the average operation time for kidney stones ranged from 18.5 min to 90 min, and the fluoroscopy time ranged from 4 s to 115 s. In addition, the ratio of kidney stone location (left/right) varies from 10/12 to 9/12. Moreover, the PCNL group used different specifications of mini-PCNL technology, and more than half of the RIRS group used 7.5 Fr F-URS. The main characteristics of each study are summarized in detail in Tables 1, 2 below.

**Table 1:** Basic characteristics of included studies.

Trials	Year	Country	Time Range	PCNL	RIRS	Sample size		
						Total	RIRS	PCNL
Bas (7)	2016	Asia-Turkey	August 2011–June 2015	Mini-PCNL	Flex-X2	81	36	45
Dogan (8)	2020	Asia-Turkey	June 2016–January 2020	Micro-PCNL	URF-P6 or URF-P7, Olympus	64	25	39
Ersoz (9)	2021	Europe-UK	2011–2018	15 Fr Mini-PCNL; 4.85 Fr Micro-PCNL; 24 Fr PCNL	7.5 Fr f-URS	76	45	31
Gamal (10)	2016	Africa-Egypt	June 2011–June 2014	Sheathless 19 Fr Mini-PCNL with 20 Fr amplatz sheath	7.5 Fr fibrooptic f-URS	42	21	21
Halinski (11)	2021	Europe-Poland	June 2015–August 2016	4.8 Fr Micro-PCNL	7.5 Fr f-URS	53	38	15
Jia (12)	2020	Asia-China	April 2016–July 2019	4.5–6.5 Fr Micro-PCNL	URF-P6, Olympus	61	25	36
Jones (13)	2020	Europe-UK	January 2010–December 2019	Mini-PCNL	Flex-X2	95	55	40
Pelit (14)	2016	Asia-Turkey	May 2013–January 2016	17 Fr Mini-PCNL	7.5 Fr f-URS	77	32	45
Resorlu (15)	2012	Asia-Turkey	January 2008–November 2011	11, 15.9, 17, and 22 Fr Mini-PCNL	—	201	95	106
Saad (16)	2015	Africa-Egypt	May 2011–February 2014	17 Fr Mini-PCNL	Flex-X2	43	21	22
Sen (17)	2017	Asia-Turkey	January 2015–April 2016	Micro-PCNL	URF-P6, Olympus	48	23	25
Wang (18)	2020	Asia-China	January 2015 and April 2016	Micro-PCNL	—	57	30	27
Zhang (19)	2021	Asia-China	June 2014–October 2019	Mini-PCNL	8-Fr/30 cm–42 cm	113	73	40

**Table 2:** Clinical characteristics of included studies.

Trials	Year	Mean age (Year)		Gender (RIRS)		Gender (PCNL)		Operation time (minutes)		Fluoroscopy time (second)		Stone size (mm)	
		RIRS	PCNL	Male	Female	Male	Female	RIRS	PCNL	RIRS	PCNL	RIRS	PCNL
Bas (7)	2016	8.39 ± 4.72	5.62 ± 4.5	—	—	—	—	—	—	—	—	12.8 ± 3.03	13.97 ± 3.46
Dogan (8)	2020	6 ± 3.63	3.08 ± 3.29	14	11	25	14	60 ± 21.25	90 ± 30	15 ± 33.75	30 ± 56.25	7 ± 0.75	11 ± 3.5
Ersoz (9)	2021	8.1 ± 5.3	8.09 ± 5.5	19	26	19	12	47.6 ± 17.6	89.9 ± 54.1	—	115 ± 95	16.01 ± 7.4	20.1 ± 10.4
Gamal (10)	2016	9.2 ± 2.5	9.2 ± 2.5	—	—	—	—	41.0 ± 8	18.5 ± 9	—	—	—	—
Halinski (11)	2021	9.6 ± 3.78	8.2 ± 3.36	—	—	—	—	—	—	—	—	12.2 ± 0	13.5 ± 0
Jia (12)	2020	4.3 ± 2.5	4.5 ± 2.7	15	10	26	10	76.3 ± 32.4	53.9 ± 22.2	—	—	14 ± 2.8	14.18 ± 3
Jones (13)	2020	9.2 ± 3.5	8.8 ± 2.13	29	26	25	15	—	—	—	—	11.4 ± 10.25	14.5 ± 3.5
Pelit (14)	2016	3.65 ± 1.95	3.71 ± 1.89	17	15	24	21	46.25 ± 9.3	85.22 ± 12.87	4.15 ± 1.98	60.88 ± 23.38	19.3 ± 4.2	21.06 ± 5.6
Resorlu (15)	2012	9.3 ± 5.2	9.6 ± 1.9	53	42	56	50	42.1 ± 15.3	76.3 ± 21.2	33.2 ± 1.46	113.7 ± 36.6	14.3 ± 3.8	23.7 ± 44.2
Saad (16)	2015	6.44 ± 4.84	6.93 ± 3.55	14	7	14	8	79.5 ± 29.4	69.8 ± 29.6	96 ± 48	186 ± 66	—	—
Sen (17)	2017	10.9 ± 3	4 ± 2.3	—	—	—	—	62.3 ± 15.3	75.1 ± 18.9	39.9 ± 15.3	115 ± 35.4	13.7 ± 3.5	12.2 ± 2.8
Wang (18)	2020	1.75 ± 0.65	1.6 ± 0.83	—	—	—	—	23 ± 5	21 ± 4	—	—	17 ± 2	16 ± 3
Zhang (19)	2021	3 ± 0.675	3.2 ± 0.55	57	16	26	14	25 ± 2.5	40 ± 10	—	—	—	—

### 3.3. Quality assessment

Based on the Cochrane Risk of Bias tool, one study was at low risk, three studies were assessed as unclear risk, and two articles were at high risk of selection bias. Of the risks of performance bias, 12 tests were low-risk, but only one clause was considered to have ambiguous risk. At the same time, the risk of detection bias was low for nine tests and uncertain for the rest. Of the attrition bias, nine articles were considered low risk, one was considered high risk, and the remaining risks were unclear. In addition, 12 articles were assessed as low-risk in the reporting bias, one was considered high-risk, and other biases are unclear. In all the trials, the risks of performance bias, detection bias, attrition bias, and reporting bias were low, while the risks of other biases were high (Supplementary Figure S1).

Each item on the Jaded scale has a score of approximately 1–5, and a trial with a score of 3 or above is identified as a high-quality trial. According to our meta-analysis, five trials received a high-quality evaluation score of 3 or above, and five studies received a low-quality evaluation score of 2 (Supplementary Table S1).

### 3.4. Primary outcome

#### 3.4.1. Analysis of the stone-free rate

Thirteen studies mentioned the stone-free rate included 1,087 patients. According to the analysis, there was a meaningful difference between the PCNL group and RIRS group [OR = 0.59; 95% CI (0.41, 0.84); *P* = 0.003] with moderate heterogeneity (*I*^2 ^= 21%). When thirteen studies were grouped according to the use of mini-PCNL or micro-PCNL, heterogeneity decreased in both groups but to different degrees. There was a meaningful difference between the RIRS group and the micro-PCNL group [OR = 0.50; 95% CI (0.29, 0.88); *P* = 0.02], indicating slight heterogeneity (*I*^2 ^= 8%). Additionally, the subgroup using mini-PCNL was not detected an overt difference between the RIRS and mini-PCNL groups [OR = 0.65; 95% CI (0.41, 1.03); *P* = 0.07], with moderate heterogeneity (*I*^2 ^= 21%). The publication is unbiased, which was assessed by Egger's test.

#### 3.4.2. Analysis of the characteristic data

##### 3.4.2.1. Analysis of age

Thirteen studies mentioned the age included 1,087 patients. According to the analysis, there was no difference between the PCNL group and RIRS group [MD = 0.06; 95% CI (−0.12, 0.24); *P* = 0.50] with high heterogeneity (*I*^2 ^= 87%). There was a significant difference between the RIRS group and the micro-PCNL group [MD = 0.62; 95% CI (0.27, 0.96); *P* = 0.0005], indicating heterogeneity (*I*^2 ^= 94%). Additionally, there is no statistical difference between the RIRS and mini-PCNL groups [MD = −0.41; 95% CI (−0.35, 0.07); *P* = 0.19], with moderate heterogeneity (*I*^2 ^= 25%). In addition, the publication is unbiased, which was assessed by Egger's test.

##### 3.4.2.2. Analysis of operation time

Ten studies mentioned the operation time included 858 patients. According to the analysis, there was an enormous difference between the PCNL group and RIRS group [MD = −8.41; 95% CI (−9.94,−6.89); *P* < 0.00001] with high heterogeneity (*I*^2 ^= 98%). There is no statistical difference between the RIRS group and the micro-PCNL group [MD = 0.17; 95% CI (−2.03, 2.37); *P* = 0.88], indicating high heterogeneity (*I*^2 ^= 93%). Additionally, there is a statistical difference between the RIRS and mini-PCNL groups [MD = −16.34; 95% CI (−18.45,−14.23); *P* < 0.00001], with high heterogeneity (*I*^2 ^= 99%). In addition, the publication is unbiased, which was assessed by Egger's test.

##### 3.4.2.3. Analysis of the stone size

Nine studies mentioned the age included 836 patients. According to the analysis, there was an enormous difference between the PCNL group and RIRS group [MD = −1.33; 95% CI (−1.90,−0.76); *P* < 0.00001] with high heterogeneity (*I*^2 ^= 83%). There was a significant difference between the RIRS group and the micro-PCNL group [MD = −1.11; 95% CI (−1.79,−0.42); *P* = 0.0001], with high heterogeneity (*I*^2 ^= 91%). Additionally, there is a statistical difference between the RIRS and mini-PCNL groups [MD = −1.87; 95% CI (−2.93,−0.81); *P* = 0.0005], with moderate heterogeneity (*I*^2 ^= 30%). In addition, the publication is unbiased, which was assessed by Egger's test.

##### 3.4.2.3. Analysis of stone location

**Figure 6:**
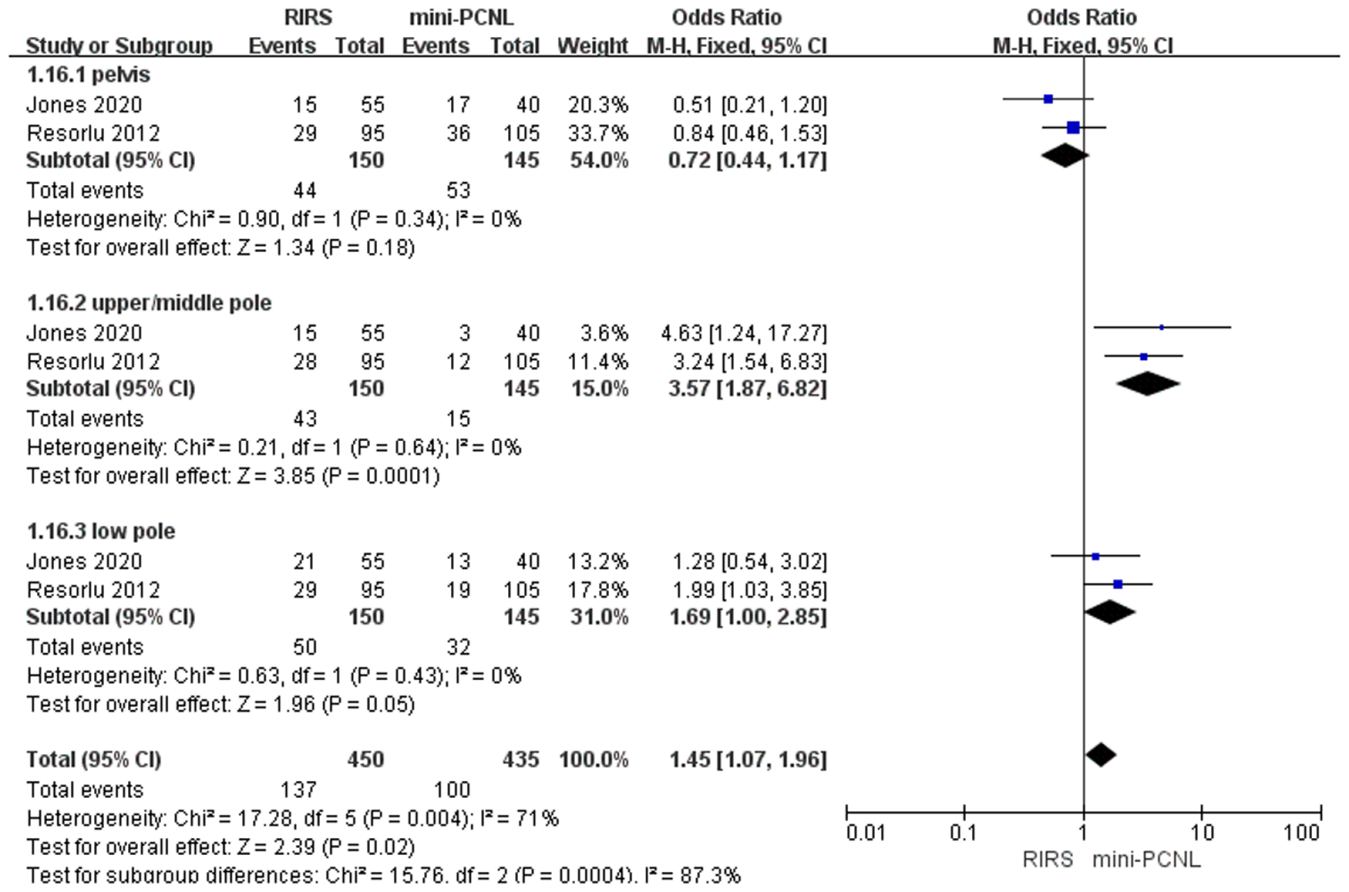
Forest plot of stone location between the RIRS group and the mini-PCNL group.

**Figure 7:**
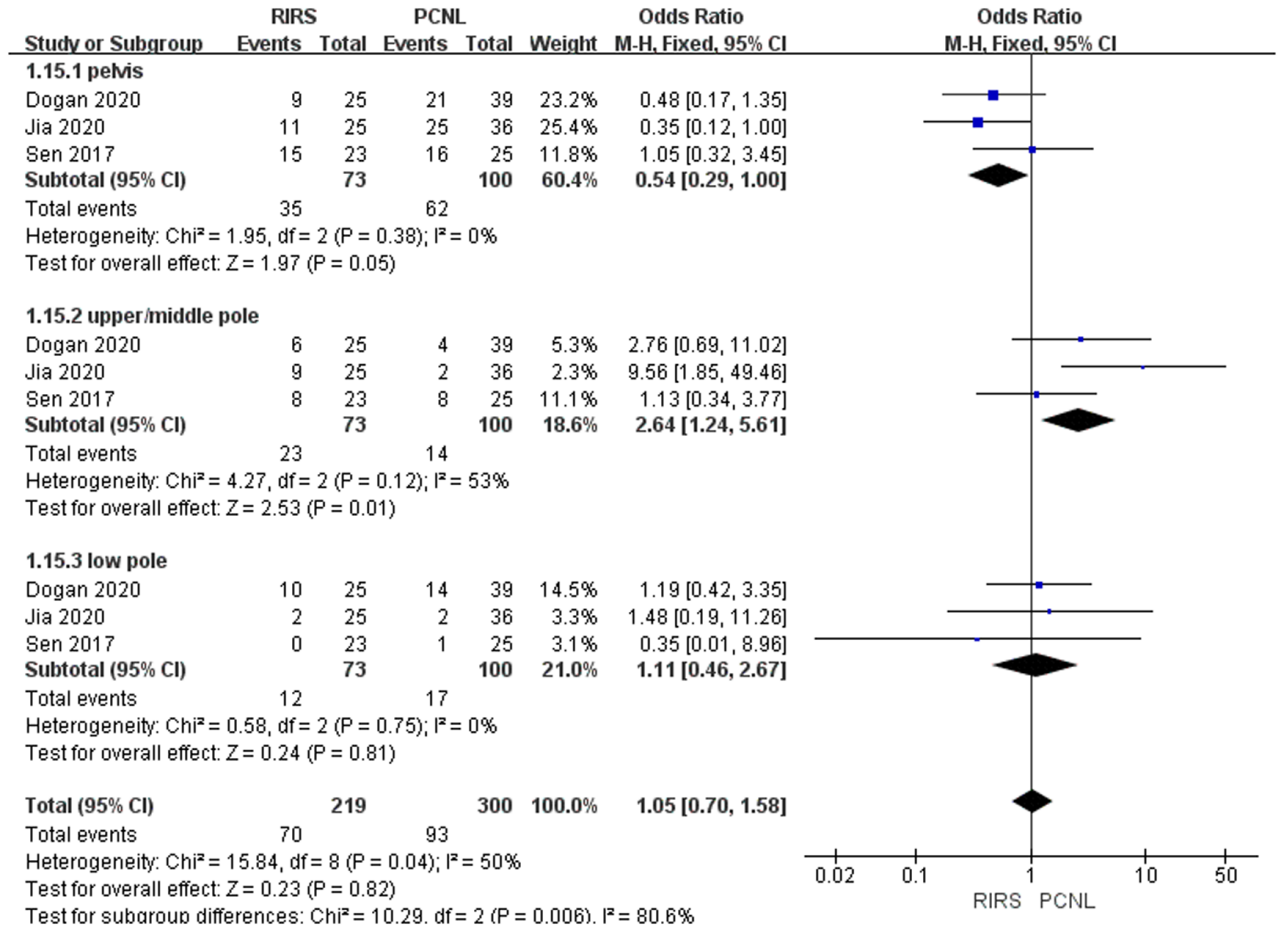
Forest plot of stone location between the RIRS group and the micro-PCNL group.

Five studies mentioned the stone location included 468 patients. There is a statistical difference between the RIRS group and the micro-PCNL group [OR = 1.05; 95% CI (0.70, 1.58); *P* = 0.70], with heterogeneity (*I*^2 ^= 50%) (Figure 7). Additionally, there was no statistical difference between the RIRS and the mini-PCNL groups [OR = 1.45; 95% CI (1.07, 1.96); *P* = 0.81], with low heterogeneity (*I*^2 ^= 71%) (Figure 6). In addition, the publication is unbiased, which was assessed by Egger's test.

#### 3.4.3. Postoperative complications

##### 3.4.3.1. Postoperative fever rate analysis

Six studies that mentioned postoperative fever (Clavien–Dindo grade I) included 441 patients. According to the analysis. There was no enormous difference between the RIRS group and PCNL group [OR = 0.96; 95% CI (0.53, 1.75); *P* = 0.91] with moderate heterogeneity (*I*^2^ = 52%). After grouping the six studies based on the usage of the mini-PCNL or micro-PCNL, the group of mini-PCNL declined completely to 0%, while that of micro-PCNL only dropped to 10%. There was a tremendous difference in the two subgroups which had conducted the micro-PCNL between the RIRS group and PCNL group [OR = 4.86; 95% CI (1.28, 18.49); *P* = 0.02] with heterogeneity (*I*^2 ^= 10%). In addition, there was no significant difference in the subgroup which had taken the mini-PCNL between the RIRS group and PCNL group [OR = 0.53; 95% CI (0.26, 1.10): *P* = 0.09] without heterogeneity (*I*^2 ^= 0%). In addition, the publication is unbiased, which was assessed by Egger's test.

##### 3.4.3.2. Analysis of Clavien–Dindo complication

**Figure 9:**
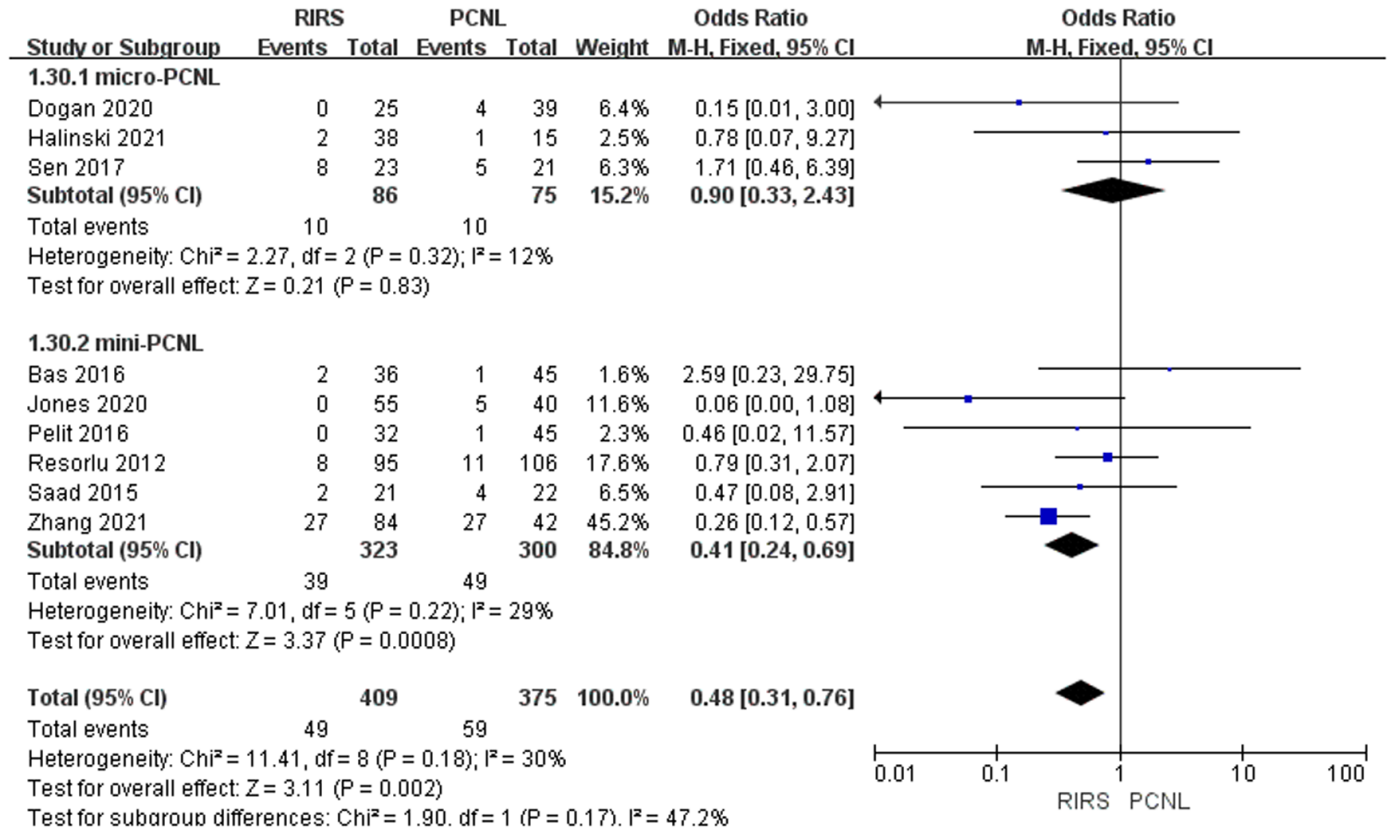
Forest plot of Clavien–Dindo grade I complication rate analysis between RIRS group and PCNL group.

Nine studies mentioned Clavien–Dindo grade I included 784 patients. According to the analysis, there was a meaningful difference between the PCNL group and RIRS group [OR = 0.48; 95% CI (0.31, 0.76); *P* = 0.002] with moderate heterogeneity (*I*^2 ^= 30%). The Micro-PCNL group dropped to 12%, while the mini-PCNL group only dropped to 29%. There was no significant difference between the RIRS group and the micro-PCNL group [OR = 0.90; 95% CI (0.33, 2.43); *P* = 0.83], indicating low heterogeneity (*I*^2 ^= 12%). Additionally, the subgroups using mini-PCNL were significantly different between the RIRS and PCNL groups [OR = 0.41; 95% CI (0.24, 0.69); *P* = 0.0008], with moderate heterogeneity (*I*^2 ^= 29%). In addition, the publication is unbiased, which was assessed by Egger's test (Figure 9).

**Figure 10:**
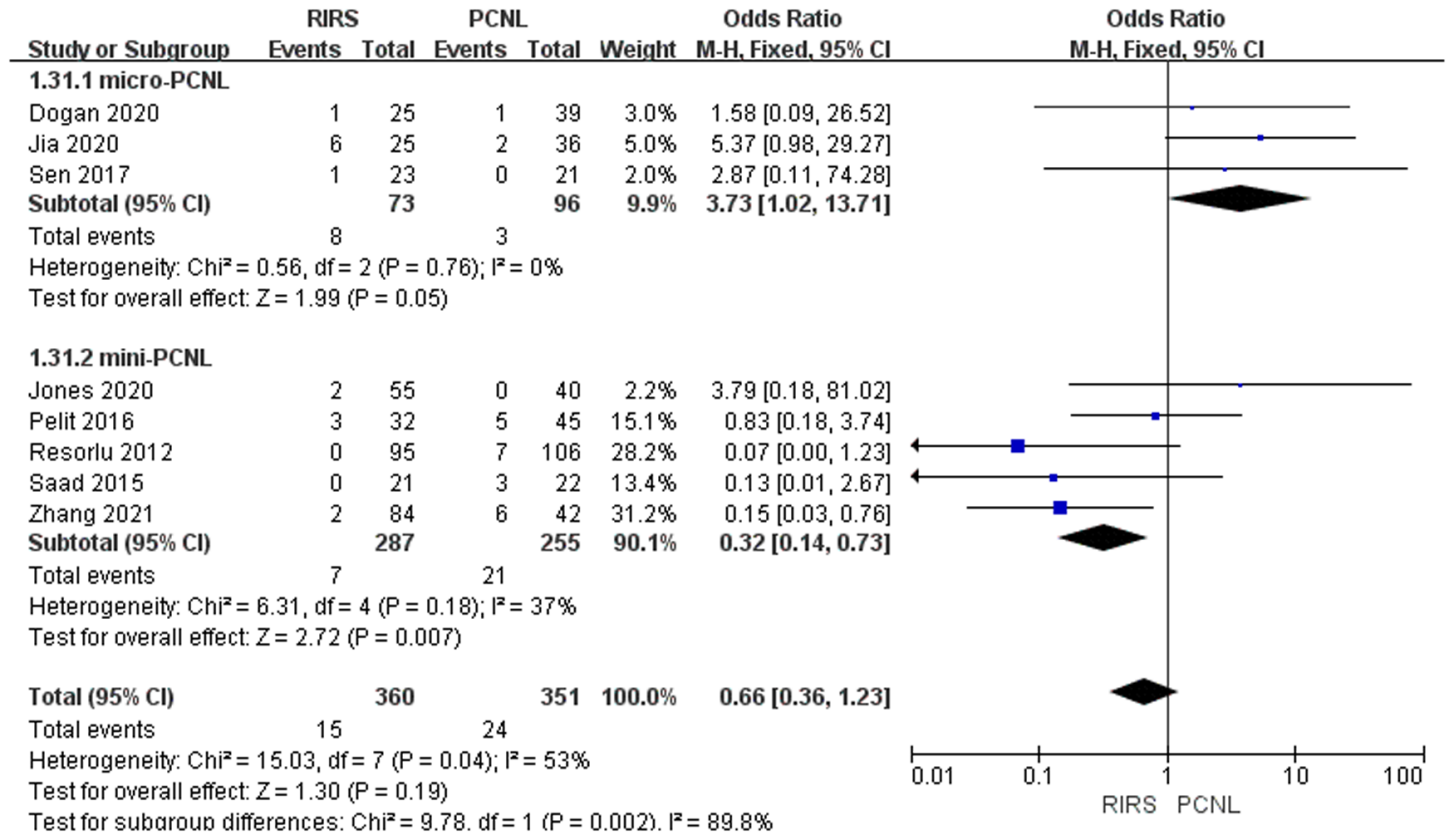
Forest plot of Clavien–Dindo grade II complication rate analysis between RIRS group and PCNL group.

Eight studies mentioned the Clavien–Dindo grade II included 711 patients. According to the analysis, there was no enormous difference between the RIRS group and PCNL group [OR = 0.66; 95% CI (0.36, 1.23); *P* = 0.19] with moderate heterogeneity (*I*^2^ = 53%). The heterogeneity in the micro-PCNL group was completely fallen to 0%, with a certain difference between the RIRS and PCNL [OR = 3.73; 95% CI (1.02, 13.71); *P* = 0.05]. At the same time, mini-PCNL was performed in the other five papers, with significant differences between the RIRS and PCNL groups [OR = 0.32; 95% CI (0.14, 0.73); *P* = 0.007] and medium heterogeneity (*I*^2 ^= 37%). In addition, the publication is unbiased, which was assessed by Egger's test (Figure 10).

**Figure 11:**
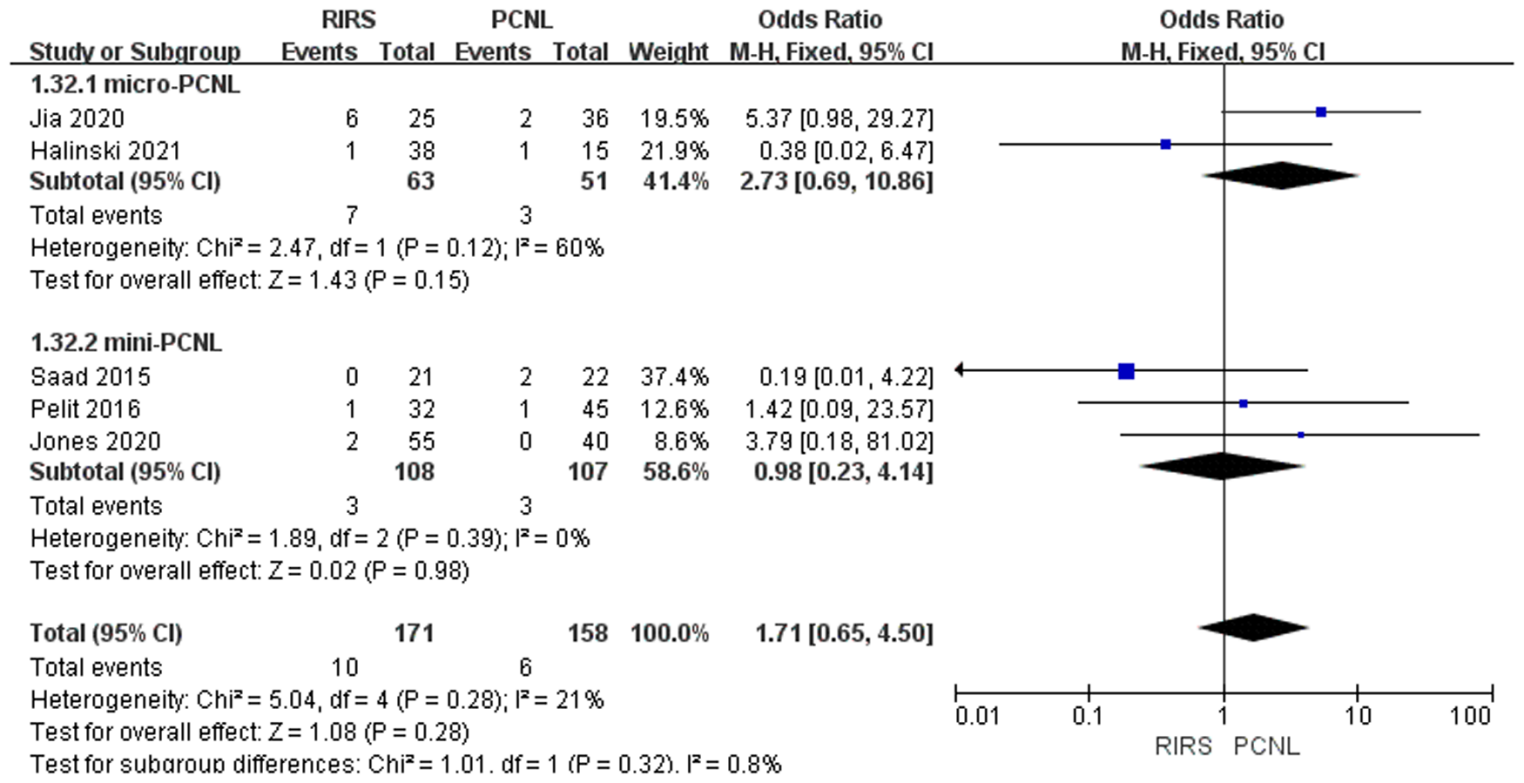
Forest plot of Clavien–Dindo grade III complication rate analysis between RIRS group and PCNL group.

Meta-analysis of Clavien–Dindo grade III between the RIRS group and PCNL group including 329 patients. The analysis concluded that there was no noticeable difference between the RIRS group and the PCNL group [OR = 1.71. 95% CI (0.65, 4.50); *P* = 0.28]with low heterogeneity (*I*^2 ^= 21%). In two studies involving micro-PCNL manipulation, Jia's article, and Halinski's article, there was no obvious difference between the RIRS group and the PCNL group [OR = 2.73; 95% CI (0.69, 10.86); *P* = 0.15]. In addition, the heterogeneity of the micro-PCNL group increased to 21%, while that of the mini-PCNL group completely decreased to 0%, and there was no significant difference between the RIRS and PCNL groups [OR = 0.98; 95% CI (0.23, 4.14); *P* = 0.98]. In addition, the publication is unbiased, which was assessed by Egger's test (Figure 11).

##### 3.4.3.3. Analysis of pooled postoperative complication rate

Twelve studies mentioned the complication rate included 1,023 patients. According to the analysis, there was a meaningful difference between the PCNL group and RIRS group [OR = 0.67; 95% CI (0.46, 0.97); *P* = 0.03] with moderate heterogeneity (*I*^2 ^= 12%). When twelve studies were grouped according to the use of mini-PCNL or micro-PCNL, heterogeneity decreased in both groups but to different degrees. There was no significant difference between the RIRS group and the micro-PCNL group [OR = 1.62; 95% CI (0.73, 3.60); *P* = 0.23], indicating heterogeneity (*I*^2 ^= 0%). Additionally, the complication rate was significantly different between the RIRS and mini-PCNL groups [OR = 0.52; 95% CI (0.34, 0.79); *P* = 0.003], with low heterogeneity (*I*^2 ^= 0%). In addition, the publication is unbiased, which was assessed by Egger's test.

## 4. Discussion

The first treatment choice for most pediatric kidney stones is ESWL (20). However, ESWL has numerous limitations, including the need for multiple sessions, general anesthesia requirements, and the shock waves' long-term effects on the developing kidney are unknown (21–23). Over the last few years, more minimally invasive surgeries have been frequently utilized as a substitute for the previous ESWL and open surgeries for kidney stones in children, such as PCNL and RIRS (24–26). Currently, the number of studies on the efficacy and prognosis of RIRS and PCNL is increasing; however, there is no agreement. Additionally, past meta-analyses that relied on a small number of earlier studies and research indicators were unable to make enough conclusions at once. So, we conducted a meta-analysis to compare the difference between RIRS and PCNL in terms of treatment means, treatment outcomes, age of patients, stone size, stone location, and postoperative complications, aiming to provide practical surgical guidance to patients.

Thirteen clinical trials were analyzed in our study, five of which were of high quality and eight of lower quality. Various postoperative complications during kidney stone surgery become a major key factor in the choice of surgical approach. We compared the effects of multiple complications in RIRS and PCNL according to our results.

**Figure 2:**
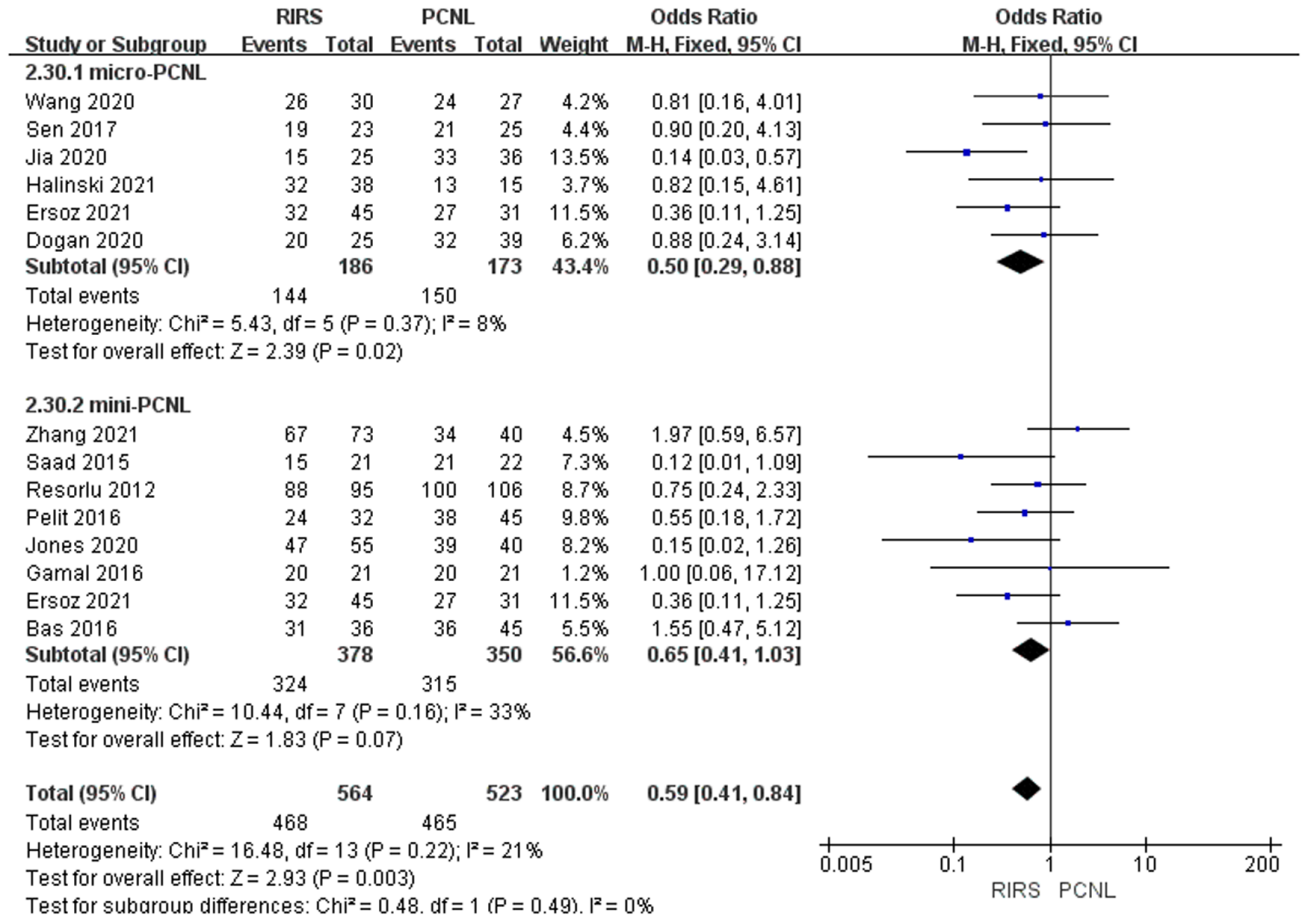
Forest plot of stone-free rate in the RIRS group and the PCNL group.

In Figure 2, our systematic review of 13 studies showed a tremendous difference in stone-free rate between the RIRS and PCNL groups. The reasons are as follows. First, PCNL usually requires multiple entrances, while RIRS always eliminates stones through a single entrance. PCNL is prone to remove large-size stones, staghorn kidney stones, and solitary kidney stones. Second, PCNL has a large dermorenal passage, fast water flow rate, and relatively high stone removal efficiency. In contrast, the low stone clearance rate of RIRS was attributed to its anatomical limitations in the treatment of stones (27). In terms of the anatomy of the kidney, such as the infundibulopelvic angle, the infundibular width, and the infundibular length make a hardship to remove the lower pole stones via RIRS (28). Besides, the insertion of the laser probe is capable of reducing the deflection of the flexible ureterorenoscopy and was not conducive for RIRS to treat the lower pole stones (27).

**Figure 3:**
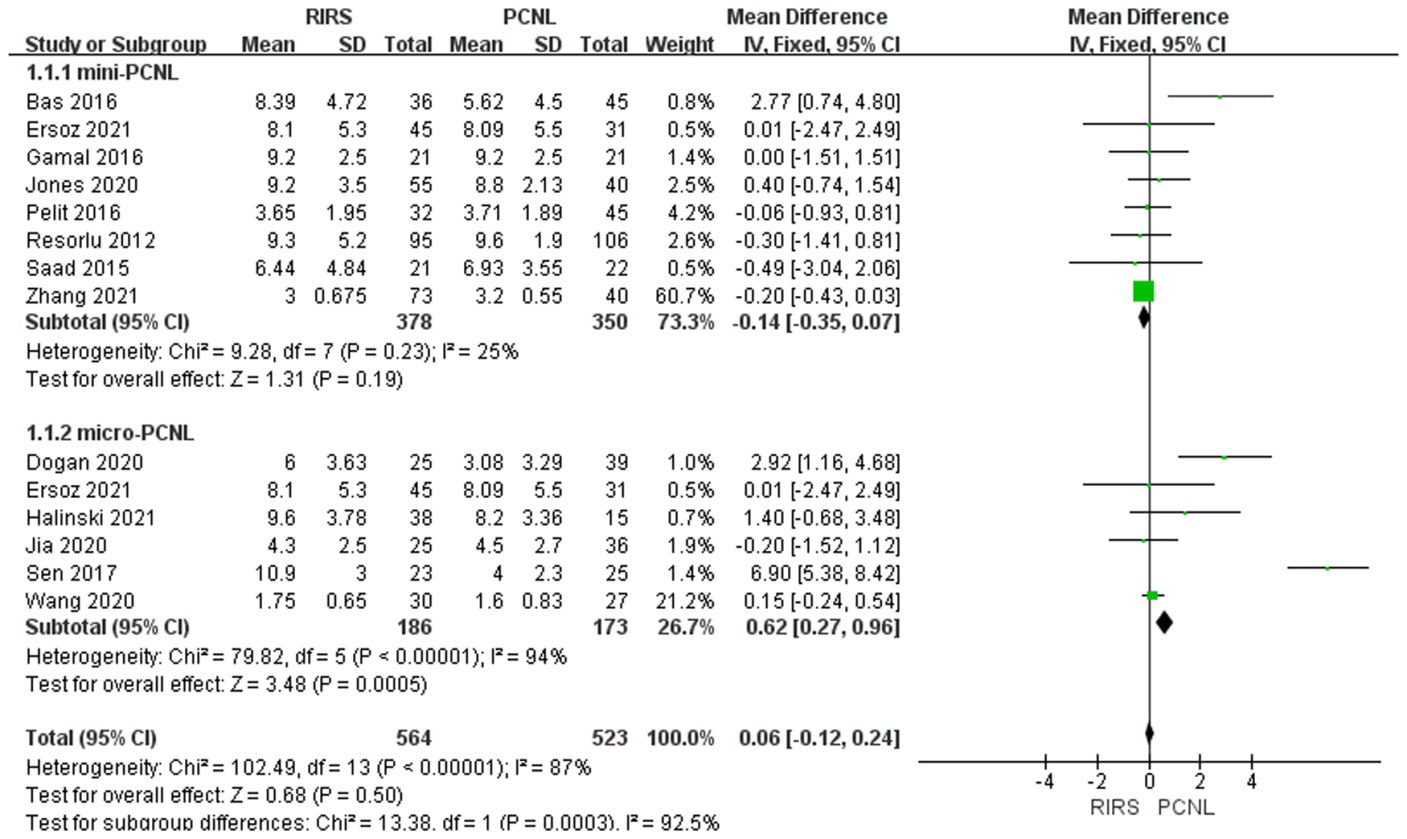
Forest plot of age between the RIRS group and the PCNL group.

**Figure 4:**
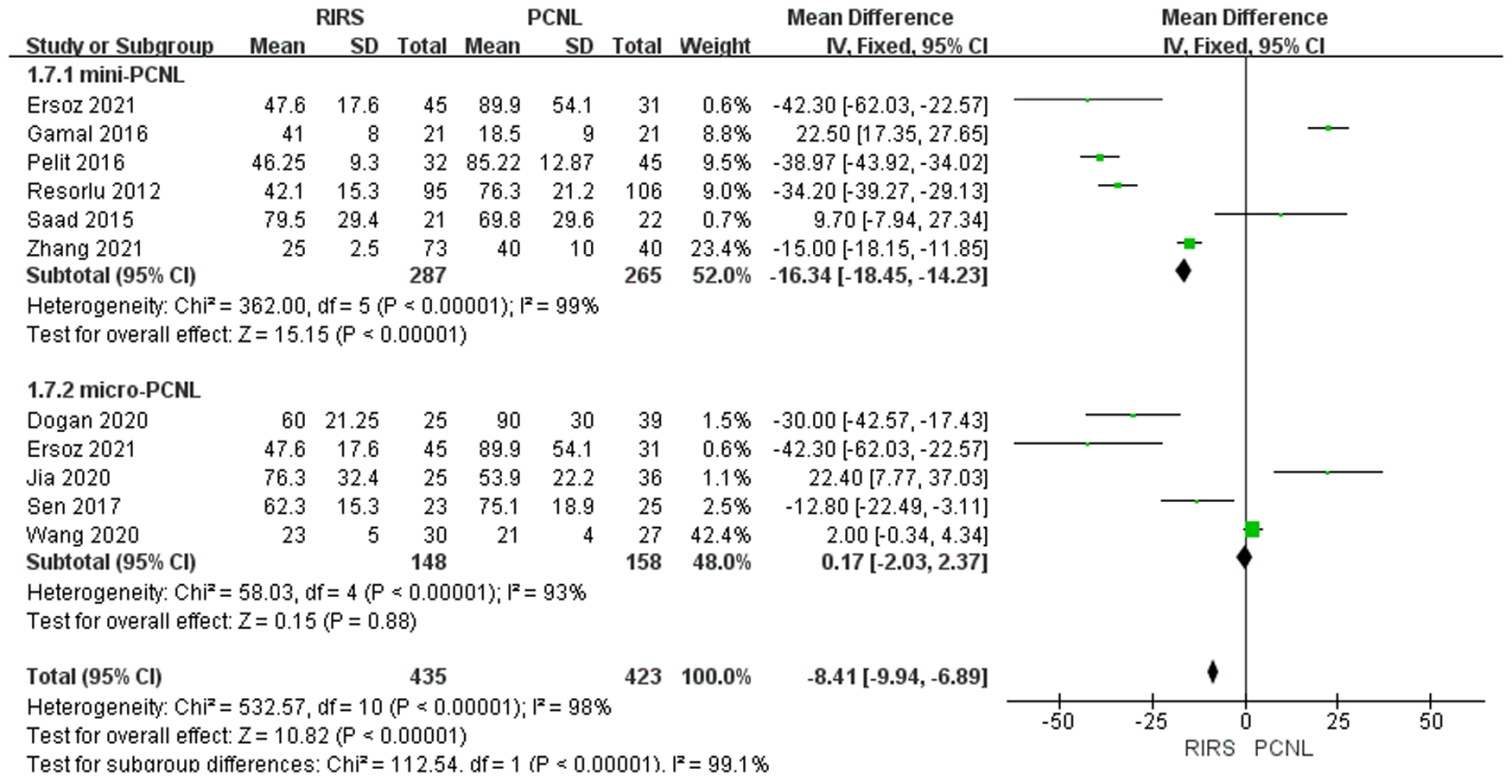
Forest plot of operation time between RIRS group and PCNL group.

**Figure 5:**
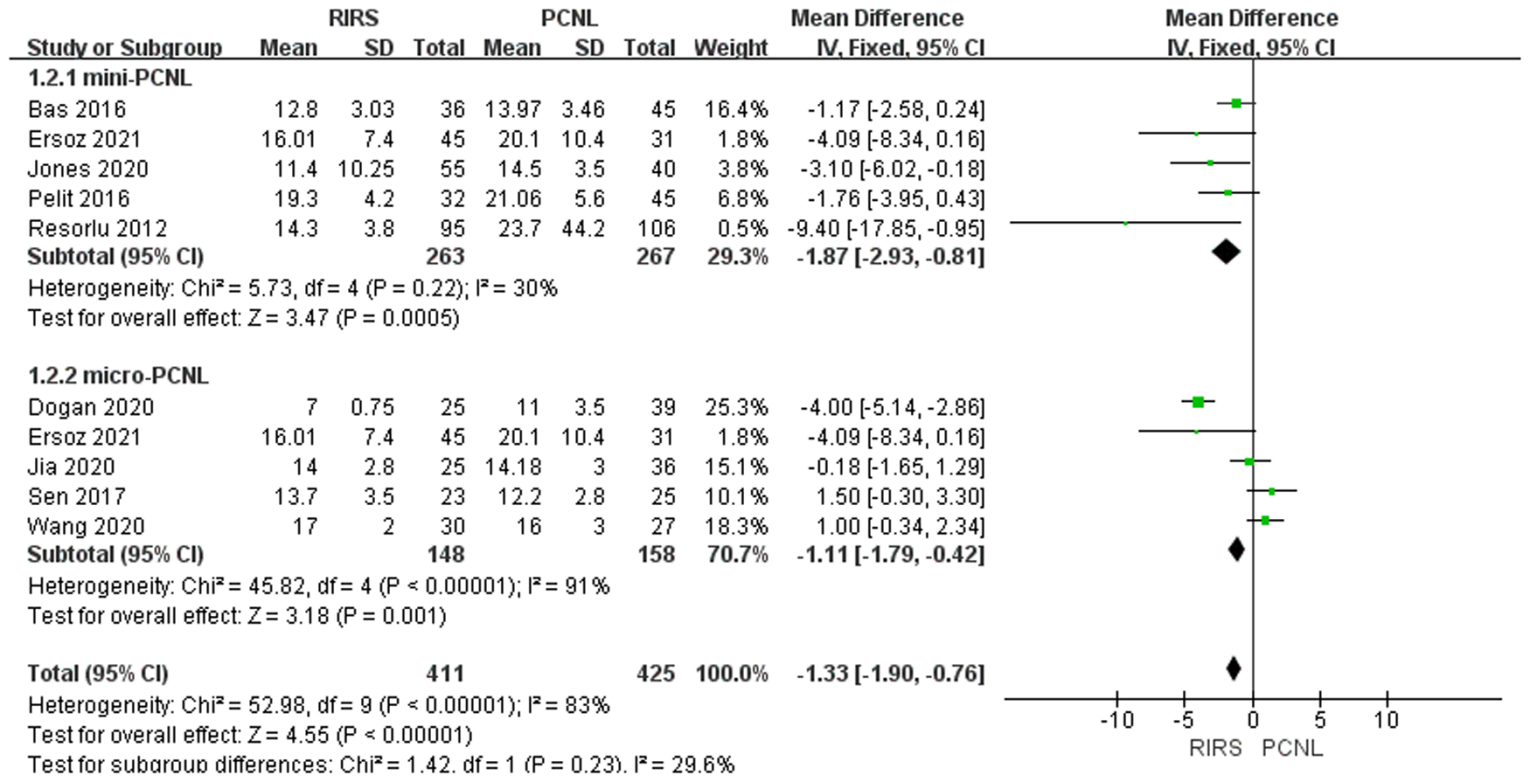
Forest plot of stone size between the RIRS group and the PCNL group.

Before analyzing the data for the complication, it is comparably important to analyze key characteristic data (eg. the mean age, the operation time, the stone size, and stone location) shown in Figures 3–7. First, we found that the mean age of patients in the RIRS group was equal to that of mini-PCNL. Of note, the mean age of children in the micro-PCNL group was significantly younger than that in the RIRS group. This result indicated that micro-PCNL may be a better clinical option for younger children, which implied age could be a risk factor for postoperative adverse effects in different PCNLs. Simultaneously, there was no exact age recommendation concerning lithotripsy in EAU guideline on pediatric urology in 2022 (29). There will be room to explore the association between the age of children and PCNL in the future. Second, we found that the mean operation time in the RIRS group was shorter than that in the mini-PCNL group. Notably, the mean operation time in the micro-PCNL group was equal to that in the RIRS group. The previous study had documented a relationship between bleeding requiring transfusion and operation time (30). Consequently, micro-PCNL and RIRS can possibly decrease severe complications. Third, about stone size, we found that the average size of stones in the RIRS group was smaller than that in the PCNL group, and ranged from 7 mm to 21.06 mm, mostly 10 mm–20 mm. This result indicated that micro-PCNL may excel in eliminating 10 mm–20 mm kidney calculi. More further studies should concentrate on the exact association between the stone size and the effects of different PCNL due to the high heterogeneity of stone sizes in included studies. Forth, the pelvis stones were mostly eliminated by PCNL, and the stones in upper/middle pole calyces were mostly disposed of by RIRS. Compared with mini-PCNL, RIRS was more frequently conducted for the stones in lower pole calyces. Most included studies were published before 2022, so our results were not consistent with the latest recommendation of the European Association of Urology (29).

**Figure 8:**
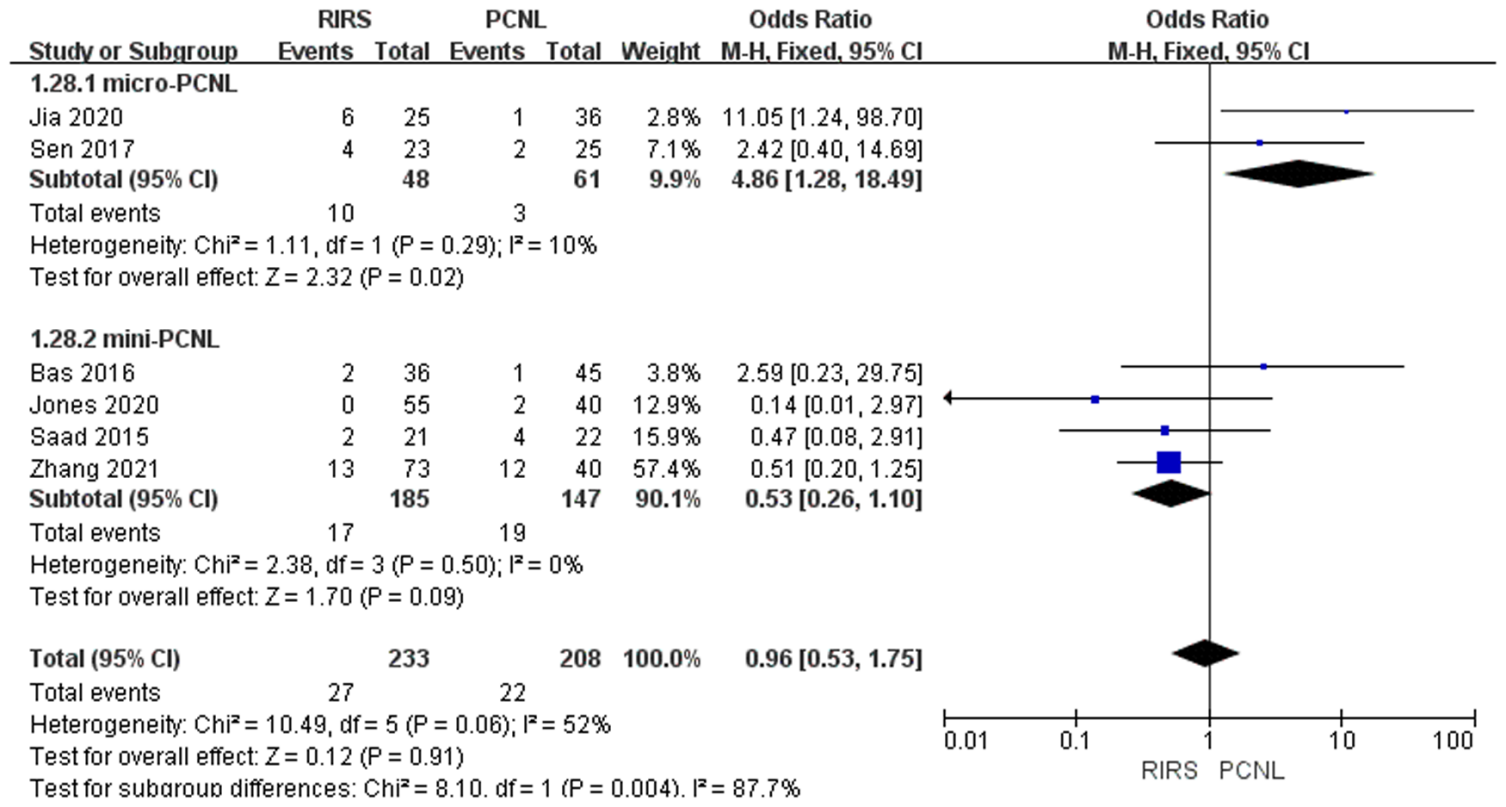
Forest plot of postoperative fever rate in the RIRS group and PCNL group.

In Figure 8, we found that the occurrence of postoperative fever in the RIRS group was better than that of the mini-PCNL group, and the treatment effect of the RIRS group was worse than that of the micro-PCNL group. Notably, the articles with fever data carried out preoperative sterile urine culture for all patients. Except for Sen's article without mentioning further antibiotic treatment, the other articles entirely used antibiotic prophylaxis or antimicrobial spectrum for treatment. The reasons that led to this result may be the following. According to Kallidonis et al., the drawbacks of mini-PCNL techniques are increased intrarenal pressure and operative time (31). High pressure favors the occurrence of urinary tract infections, which in turn causes fever. What is more, compared with RIRS, mini-PCNL has been bound up with more significant complications such as postoperative bleeding and organic injury, due to its invasive skin puncture (32). Additionally, it is also possible that fluoroscopy-guided renal access increases the radiation exposure time and higher risk of iatrogenic visceral injury, thus making complications more lethal, supposing a larger size PCNL is conducted (32).

**Figure 12:**
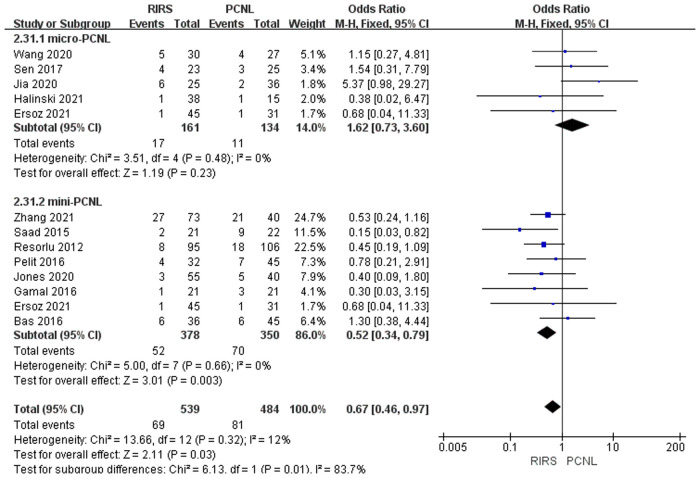
Forest plot of pooled postoperative complication between the RIRS group and PCNL group.

We summarized the Clavien–Dindo grade I, II, and III complication data for the combined analysis from the perspective of efficacy and safety, respectively, as shown in Figures 9–11. For Clavien–Dindo grade I, as is shown in Figure 9, the complication rate was lower in the RIRS group than in the mini and micro-PCNL groups. For Clavien–Dindo grade II in Figure 10, micro-PCNL had better therapeutic effects than the RIRS group. For Clavien–Dindo grade III in Figure 11, the difference in complication rates between the RIRS group and the PCNL group was not statistically significant. When it comes to pooled complication rate in Figure 12, the mini-PCNL had statistically better effects. Collectively, the micro-PCNL is possibly prone to a decline in the complication rate. The reasons for these results are complex, but it is important that RIRS clear kidney stones through natural pathways, while PCNL, especially the mini-PCNL, cleans the stones by establishing a large incision. Also, it is worth noting that there are at least half of the children are not capable to obtain retrograde process, who should be left stents to enlarge the orifice and receive a second session, thus possibly ascending the complication rate (33).

We find the following conclusions when we compare our research to other meta-analyses already done. First and foremost, Lu et al. suggested that PCNL therapy had a higher stone-free rate for pediatric patients. Nevertheless, they included only four studies for analysis which is not enough, and there was no statistical significance in complication rate and SFR (34). Zhao et al. concluded that more minimally invasive PCNL is the optimal procedure for managing kidney stones in children by contrasting the results of SWL, RIRS, and PCNL at different degrees of minimal invasiveness. This conclusion is essentially the same as in the meta-analysis by Sharma et al. on adult kidney stones. However, these two studies included fewer RCTs, and their findings need more high-quality RCTs for validation (35, 36). In the study by Gu et al., in contrast to micro-PCNL, RIRS had a higher SFR and more hemoglobin loss, and no differences were identified in hospitalization, fluoroscopy durations, or complication rates (37). However, in the study of Chen et al., compared with PCNL, without compromising the stone-free rate or the length of surgery, RIRS has a superior prognosis when treating pediatric patients with upper urinary stones. Similarly, their experiments did not include enough RCTs. Moreover, Chen's study ignored the high heterogeneity of stone-free rate, operative time, hospital stay, and fluoroscopy time (38). In contrast, our study included sufficient RCTs and provided a more comprehensive and objective illustration of the problem.

There are several limitations to our study. First of all, only four included researches are of high quality via the jadad assessment, and some studies are retrospective, indicating that our study's authenticity and accuracy may be affected. Second, although thirteen studies were included, there were not a large amount of data being extracted. More parameters should be analyzed to determine children's superiority over RIRS or PCNL. Third, some uncontrolled factors may affect the outcomes, such as different races, different levels of surgical experience, and diet. Fourth, a significant limitation was the differences in mean stone size in our included studies, most of which are 10 mm–20 mm, while mean stone sizes are more extensive than 20 mm or more minor than 10 mm in some studies. Fifth, our study did not conduct subgroups to analyze the relationship between stone position, stone constituent, and efficacy of the surgeries. Ultimately, our meta-analysis does not find the absolute superiority of PCNL and RIRS in contemporary pediatric patient cohorts due to poor cases.

## 5. Conclusion

First and foremost, though the PCNL group had a significantly larger stone size, the mini and micro-PCNL had a higher stone-free rate than the RIRS group. Also, when performing micro-PCNL, the operation time was shorter than other surgery. Notably, considering the Dindo–Clavien classifications and overall complication rate, micro-PCNL tended to induce fewer postoperative complications. Collectively, we recommend micro-PCNL as a better therapeutic option for 10–20 mm pediatric kidney calculi.

## Data Availability

The original contributions presented in the study are included in the article/**Supplementary Material**, further inquiries can be directed to the corresponding author.
