# Supplementary figures and images for: A meta-analysis: retrograde intrarenal surgery vs. percutaneous nephrolithotomy in children
Source: Front Pediatr. 2023 May 2;11:1086345. doi: 10.3389/fped.2023.1086345 (PMC10185757; doi:10.3389/fped.2023.1086345)

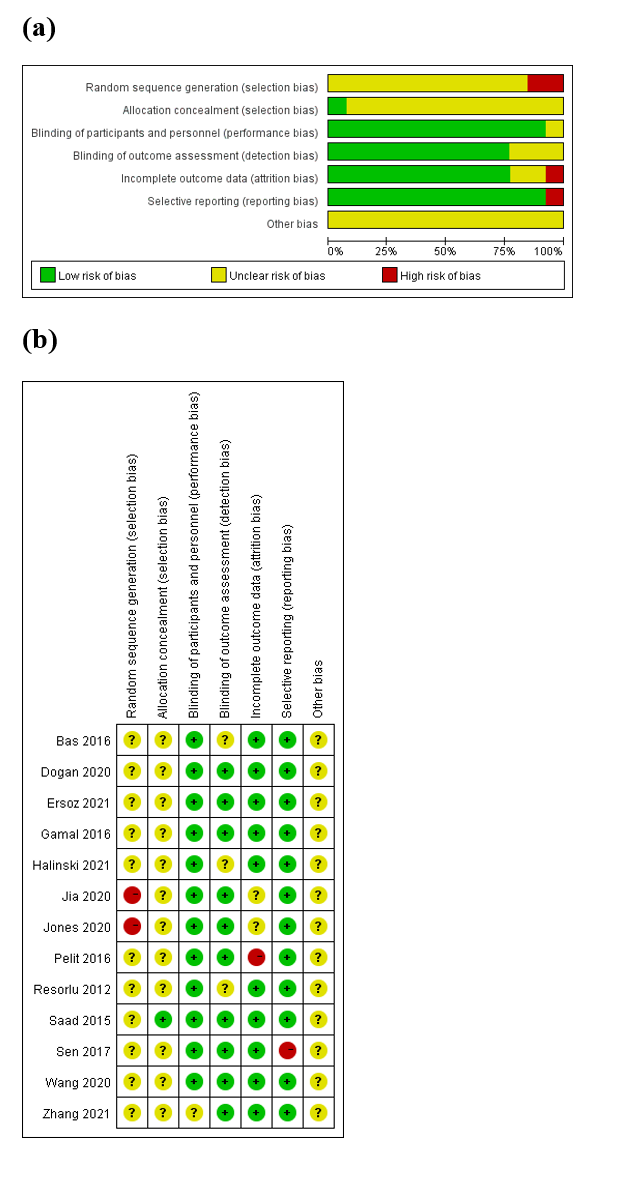

Supplement: Supplementary file 1 [file Datasheet1.zip › Figure S1.png]

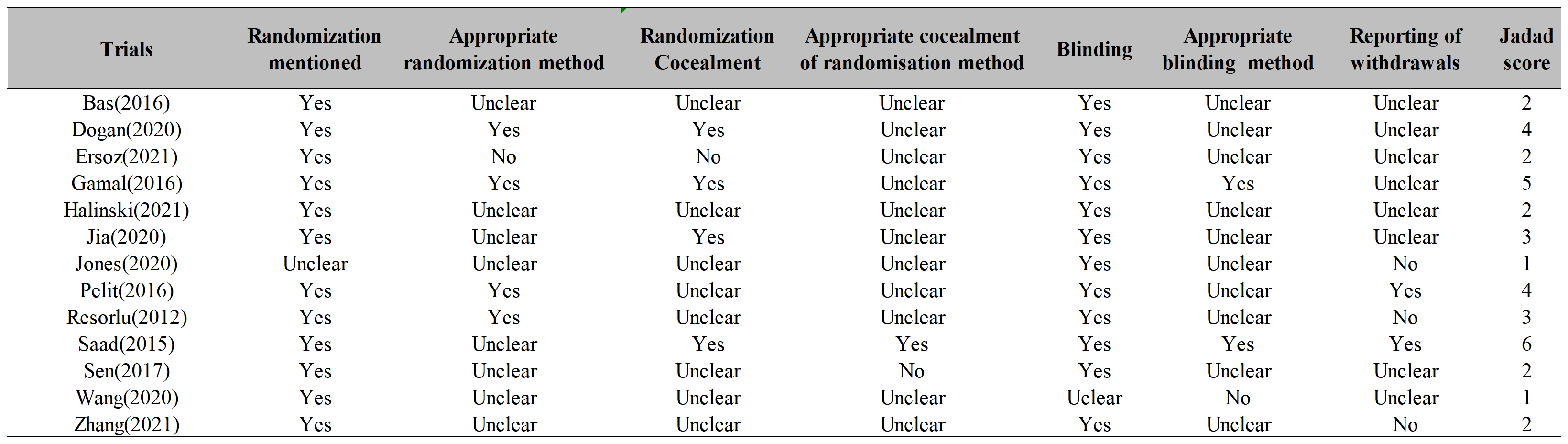

Supplement: Supplementary file 1 [file Datasheet1.zip › Table S1.png]
